# 0736. Role of _C_AMP in PAF-induced intestinal endo-and epithelial dysfunction

**DOI:** 10.1186/2197-425X-2-S1-P58

**Published:** 2014-09-26

**Authors:** I Lautenschläger, K Zitta, J Sarau, H Dombrowsky, YL Wong, M Albrecht, S Uhlig, I Frerichs, N Weiler

**Affiliations:** Dept. Anaesthesiology and Intensive Care Medicine, University Medical Centre Schleswig-Holstein, Kiel, Germany; Priority Area Asthma and Allergies, Leibniz Centre for Medicine and Biosciences, Borstel, Germany; Dept. of Pharmacology and Toxicology, RWTH, Aachen, Germany

## Introduction

Platelet activating factor (PAF) induces vascular barrier breakdown and intestinal failure that contribute to the development of sepsis. The exact cellular mechanisms are not well understood.

## Objectives

We aim to analyse the role of cAMP in PAF-induced intestinal endo- and epithelial dysfunction.

## Methods

An isolated model of the rat small bowel (1) was used. Intestines were stimulated with a 0.5 nmol PAF bolus via the mesenteric artery alone (PAF, n=5) or after pretreatment with IBMX (100µM) and forskolin (0.5µM) for 20 min (PAF+PDE/AC, n=4) to increase intracellular cAMP by inhibition of phosphodiesterase (PDE) and stimulation of adenylate cyclase (AC). The pressure responses, the vascular fluid loss and the transfer of FITC-labeled vascular dextran were monitored. cAMP was measured in PAF stimulated and untreated intestines (n=5) 3 min after PAF-stimulation or at the equivalent time point.

## Results

The maximal pressure amplitude (ΔPmax), the time delay to achieve ΔPmax (tdΔPmax), the vascular volume loss (Vloss) as well as the macromolecule transfer to the lymph (FITClym) and to the lumen (FITClum) were reduced significantly by inibition of PDE and stimulation of AC [Table 1].

**Table 1:** Table 1

	PAF	PAF+PDE/AC	
**ΔPmax** (mmHg)	30.2±4.0	14.7±1.4	p<0.05
**tdΔPmax** (min)	1.45±0.15	0.77±0.11	p<0.05
**Vloss** (ml)	19.2±5.7	3.6±0.7	p<0.05
**FITClym** (mg/15min/g ^Ψ^ )	0.407±0.078	0.026±0.011	p<0.05
**FITClum** (mg/15min/g ^Ψ^ )	0.671±0.169	0.013±0.010	p<0.05
^Ψ^ , *dry weight*			

cAMP levels in control and PAF treated intestines were comparable (cAMPCON 4.99±1.76 nM vs cAMPPAF 4.98±0.88 nM, p>0.1).

## Conclusions

While drugs that increase the intracellular cAMP concentration protect the intestine from PAF-induced endo- and epithelial dysfunction, all the cellular effects of PAF can not be explained by a deprivation of cAMP in the intestine.
